# Supplementary material for: Bounded distributions place limits on skewness and larger moments
Source: PLoS One. 2024 Feb 9;19(2):e0297862. doi: 10.1371/journal.pone.0297862 (PMC10857697; doi:10.1371/journal.pone.0297862)
Supplement: S1 Appendix — (PDF) [file pone.0297862.s001.pdf]

Appendix A: Discreet Distribution Decomposition

Rohatgi and Székely derived the result that any discrete distribution with mean μ can be decomposed into a sum of bidisperse distributions, all with mean μ [9]. Their derivation is terse, so we rederive the result in this Appendix with a slightly lengthier presentation.

First, consider a discrete distribution $P(x)$ where x can take values a_i with probability p_i for $1 \leq i \leq n$, $\Sigma_i p_i = 1$, and with mean $\Sigma_i p_i a_i = \mu$. Replace a_n and a_{n-1} by

$$a'_{n-1} = \frac{p_{n-1}}{p_{n-1} + p_n} a_{n-1} + \frac{p_n}{p_{n-1} + p_n} a_n \tag{1}$$

which occurs with probability $p'_{n-1} = p_{n-1} + p_n$. This is now a new distribution with mean μ and one fewer value. This can be repeated until one ends with a final distribution that takes on three discrete values, a_1, a_2, and a'_3 with probabilities p_1, p_2, and p'_3.

If we have a tridisperse distribution with three discrete values (a_1, a_2, a_3), with probabilities (p_1, p_2, p_3) and mean μ, we can decompose this into the sum of two bidisperse distributions as follows. Without loss of generality, assume $a_1 < \mu$ and $a_2 \leq \mu$. Then the first bidisperse distribution has values (a_1, a_3) with probabilities $p'_1 = \frac{a_3 - \mu}{a_3 - a_1}$ and $p'_3 = \frac{\mu - a_1}{a_3 - a_1}$, and similarly for the second distribution with values (a_2, a_3). Sampling the first distribution with probability p_1/p'_1 and the second with probability p_2/p'_2 recovers the original tridisperse distribution.

Now consider the distribution with four discrete values (a_1, a_2, a_3, a_4) and the related distribution (a_1, a_2, a'_3) formed using Eq (A.1). The latter can be decomposed as a sum of two bidisperse distributions, as just demonstrated. This then provides a scheme to reduce the four-valued distribution to a sum of two three-valued distributions, one of which eliminates a_1 and the other which eliminates a_2. That is, the probability of finding a'_3 in each of the two bidisperse distributions is used to determine the new probabilities of finding a_3 and a_4 in the two tridisperse distributions. Proceeding by induction, each distribution with n distinct a_i values can be decomposed into two distributions of $n - 1$ distinct values, ultimately reducing down to a sum of bidisperse distributions.
