# Supplementary material for: Bounded distributions place limits on skewness and larger moments
Source: PLoS One. 2024 Feb 9;19(2):e0297862. doi: 10.1371/journal.pone.0297862 (PMC10857697; doi:10.1371/journal.pone.0297862)
Supplement: S2 Appendix — (PDF) [file pone.0297862.s002.pdf]

A Appendix B: Derivation of Eq (43)

We wish to show that Eqs (41) and (43) are equivalent expressions for D_n for a bidisperse distribution. It is easiest to start with the end result and work backwards: Eq (43) is

$$D_n = \sum_{i=1}^{n-1} (-1)^{(n-i+1)} (z)^{(2i-n)} \tag{1}$$

$$= \sum_{i=1}^{n-1} (-1)^{(n-i+1)} \left(\frac{1-a/\mu}{\delta} \right)^{(n-2i)} \tag{2}$$

where a can represent either a_+ or a_-. We will begin by examining the term with a, μ, and δ and work to express it in terms of η and q. We will initially assume $a = a_-$ and use Eq (6) to express a_- in terms of η, μ, and q; and likewise we will use Eq (9) to express δ in terms of those same variables. This leads to

$$\frac{1-a_-/\mu}{\delta} = \frac{\mu - a_-}{\mu\delta} \tag{3}$$

$$= \frac{\mu - [\mu/(1-q+\eta q)]}{\mu(\eta-1)\sqrt{q-q^2}/(1-q+\eta q)} \tag{4}$$

$$= \frac{1-q+\eta q-1}{(\eta-1)\sqrt{q-q^2}} \tag{5}$$

$$= \frac{q}{\sqrt{q(1-q)}} = \left(\frac{q}{1-q} \right)^{1/2}. \tag{6}$$

We can put this in to Eq (B.2) to give

$$D_n = \sum_{i=1}^{n-1} (-1)^{(n-i+1)} \left(\frac{q}{1-q} \right)^{(n/2)-i} \tag{7}$$

$$= (-1)^{n+1} \left(\frac{q}{1-q} \right)^{n/2} \left[\sum_{i=1}^{n-1} (-1)^i \left(\frac{1-q}{q} \right)^i \right] \tag{8}$$

where now the summation is simply a finite geometric sum. The sum can be evaluated as

$$\sum_{i=1}^{n-1} \left(\frac{q-1}{q} \right)^i = \frac{\left(\frac{q-1}{q} - \left(\frac{q-1}{q} \right)^n \right)}{1 - \frac{q-1}{q}} \tag{9}$$

$$= (q-1) - q \left(\frac{q-1}{q} \right)^n. \tag{10}$$

Putting this in to Eq (B.8), recognizing that $(q-1)^n = (-1)^n (1-q)^n$, and distributing the leading factor of $(-1)^{n+1}$, we get

$$D_n = \left(\frac{q}{1-q} \right)^{n/2} \left[(-1)^n (1-q) + q^{1-n} (1-q)^n \right], \tag{11}$$

and this can be simplified to Eq (41).

The starting point we used above was Eq (B.6):

$$\frac{1 - a_-/\mu}{\delta} = \left(\frac{q}{1-q}\right)^{1/2}.$$

If instead one focuses on a_+, the equivalent result is

$$\frac{1 - a_+/\mu}{\delta} = -\left(\frac{1-q}{q}\right)^{1/2}. \tag{12}$$

Given that Eq (B.1) is unchanged when replacing $z \to -(1/z)$, the derivation holds whether using a_+ or a_-. Thus, the 'a' in Eq (B.2) is valid for either meaning of a, and we have shown that Eqs (41, 43) are equivalent.
